# Linear Dichroism of the Optical Properties of SnS and SnSe Van der Waals Crystals

**DOI:** 10.1002/smll.202410903

**Published:** 2025-02-21

**Authors:** Agata K. Tołłoczko, Jakub Ziembicki, Miłosz Grodzicki, Jarosław Serafińczuk, Marcin Rosmus, Natalia Olszowska, Sandeep Gorantla, Melike Erdi, Seth A. Tongay, Robert Kudrawiec

**Affiliations:** ^1^ Department of Semiconductor Materials Engineering Wrocław University of Science and Technology Wybrzeże Wyspiańskiego 27 Wrocław 50‐370 Poland; ^2^ Łukasiewicz Research Network – PORT Polish Centre for Technology Development Stabłowicka 147 Wrocław 54066 Poland; ^3^ Solaris National Synchrotron Radiation Centre Jagiellonian University Czerwone Maki 98 Kraków 30‐392 Poland; ^4^ School for Engineering of Matter Transport and Energy Arizona State University Tempe AZ 85287 USA

**Keywords:** anisotropy, linear dichroism, optical absorption, photoemission spectroscopy, tin selenide, tin sulphide, Van der Waals crystals

## Abstract

Tin monochalcogenides SnS and SnSe, belonging to a family of Van der Waals crystals isoelectronic to black phosphorus, are known as environmentally friendly materials promising for thermoelectric conversion applications. However, they exhibit other desired functionalities, such as intrinsic linear dichroism of the optical and electronic properties originating from strongly anisotropic orthorhombic crystal structures. This property makes them perfect candidates for polarization‐sensitive photodetectors working in near‐infrared spectral range. A comprehensive study of the SnS and SnSe crystals is presented, performed by means of optical spectroscopy and photoemission spectroscopy, supported by ab initio calculations. The studies reveal the high sensitivity of the optical response of both materials to the incident light polarization, which is interpreted in terms of the electronic band dispersion and orbital composition of the electronic bands, dictating the selection rules. From the photoemission investigation the ionization potential, electron affinity and work function are determined, which are parameters crucial for the design of devices based on semiconductor heterostructures.

## Graphical abstract

SnS and SnSe van der Waals crystals exhibit strong anisotropy of the optical properties, which makes them perfect candidates for applications in polarization‐sensitive photodetection. The phenomenon is investigated by means of optical spectroscopy and explained in terms of  its origins in the electronic band structure.

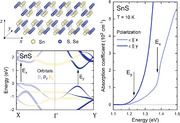

## 1 Introduction

**Figure 1:**
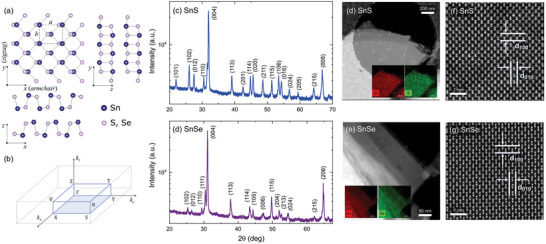
a) Schematic illustration of the crystal structure created with VESTA software,^[^ ^48^ ^]^ b) the first Brillouin zone of the reciprocal orthorhombic structure. The results of powder XRD characterization for c) SnS and d) SnSe. Images of the crystals acquired by means of HAADF‐STEM, visualizing the d) SnS and e) SnSe flakes, with EDS maps in the insets, confirming the homogenous distribution of Sn and S or Se atoms. High‐resolution HAADF‐STEM images of the atomic structure of f) SnS and g) SnSe, with indicated spacings between crystal (100) and (010) planes, corresponding to *a* and *b* lattice parameters, respectively.

The unique layered structure and possibility of obtaining atomically thin flakes make van der Waals (vdW) crystals^[^ ^1^, ^2^ ^]^ perfect candidates for applications in electronics and optoelectronics, such as photodetectors, solar cells, and light emitters. An interesting class of devices are polarization‐sensitive photodetectors, as their responsivity depends on the incident light polarization. Such technology can be exploited for the detection of light polarization changes after traveling through a birefringent medium, including various solids, and liquid crystals, but also biological systems, such as protein solutions.^[^ ^3^ ^]^ In the case of the latter, the measured polarization angle shift may allow us to determine the presence of certain proteins in a sample, which is extremely important in diagnostics. Unfortunately, the state‐of‐the‐art polarization‐sensitive photodetectors are rather complex, as they demand the integration of multiple optical components. The design, however, can be significantly simplified by exploiting the materials with intrinsic anisotropy of the optical properties.^[^ ^4^, ^5^, ^6^ ^]^ Among vdW crystals, such properties were observed for black phosphorus (BP)^[^ ^6^, ^7^, ^8^ ^]^ and its binary analogs, group IV monochalcogenides (MX, where M = Ge, Sn, and X = S, Se).^[^ ^9^, ^10^, ^11^, ^12^, ^13^, ^14^, ^15^, ^16^ ^]^ Since MXs exhibit stability in atmospheric conditions superior to BP,^[^ ^9^, ^17^ ^]^ they are more suitable for most applications, including photodetection. The origin of the anisotropy is the structure of the materials. Similarly to BP, MXs crystalize in a distorted orthorhombic structure (space group *Pnma*, no. 62) with strong in‐plane anisotropy of the atomic arrangement, as schematically presented in **Figure** **1**a. Along one of the axes, often referred to as the *armchair* direction, the structure is puckered, while a ladder‐like pattern is observed in the orthogonal direction, *zigzag*. The anisotropy of the crystal lattice induces directionality of the electronic band dispersion, which then determines the dielectric function and the optical properties. For the considered 1:1 stoichiometry, the metal atom is in the +2 oxidation state and forms three bonds with chalcogen atoms and a lone electron pair in the tetragonal coordination.^[^ ^18^ ^]^ The presence of stereochemically active lone pairs is also known to reduce the ionization potential of a material^[^ ^19^, ^20^, ^21^ ^]^ and influence the photoconversion efficiency, as in the case of metal‐halide perovskites.^[^ ^22^, ^23^ ^]^

Among the MX family, tin monochalcogenides are characterized by relatively narrow indirect bandgaps in the near‐infrared spectral range (1.1 and 0.9 eV for SnS and SnSe, respectively),^[^ ^24^, ^25^ ^]^ high absorption coefficient, and intrinsic *p*‐type conductivity, enhanced by the presence of native acceptor defects,^[^ ^26^, ^27^, ^28^, ^29^, ^30^ ^]^ making them perfect candidates for infrared polarization‐sensitive photodetectors. Apart from optoelectronics, SnX crystals pose vast potential for applications as thermoelectric materials,^[^ ^31^, ^32^, ^33^, ^34^ ^]^ extremally desired in the era of growing energy demand. For SnSe, Zhao et al.^[^ ^31^ ^]^ reported an unexpectedly high thermoelectric Figure of merit of 2.6, exceeding the performance of typical state‐of‐the‐art Pb‐based materials.^[^ ^35^ ^]^

In this work, we investigate the linear dichroism of the optical properties of SnS and SnSe by means of optical spectroscopy and search for its origins in the electronic band structure, studied by combined density functional theory (DFT) calculations and photoemission spectroscopy. We discuss the influence of the stereochemical activity of the lone electron pairs on the energies of the electronic bands with respect to the vacuum level, a factor crucial for the engineering of semiconductor heterostructures and metal contacts.^[^ ^27^, ^36^, ^37^, ^38^, ^39^ ^]^ The considerations also include other materials from the MX family, allowing us to identify and visualize chemical trends exhibited by crystals with different compositions, thereby providing a broader context for understanding their electronic and optical properties. These findings provide an in‐depth understanding of the mechanisms responsible for the observed phenomena at the fundamental level and highlight the empirical implications of the selection rules.

## 2 Results and Discussion

### 2.1 Structural Characterization

SnS and SnSe crystalize in a distorted orthorhombic phase, schematically presented in Figure 1a. In Figure 1b the first Brillouin zone (BZ) of the reciprocal space is illustrated, with marked high‐symmetry points. It is important to note that in the literature and crystallographic databases, different notations for labeling the crystallographic directions are used, resulting from the arbitrary choice of the orientation of the orthorhombic unit cell. To avoid confusion and maintain consistency with our previous works^[^ ^14^, ^40^ ^]^ (especially regarding angle‐resolved photoemission measurements, ARPES,^[^ ^40^ ^]^ where reciprocal direction *k_z_* is typically associated with the axis perpendicular to the probed plane), we assume the following assignation of the crystallographic directions: the [100] direction (*x*‐axis, lattice parameter *a*) corresponds to the in‐plane direction with puckered atomic chain (*armchair*). The [010] direction (*y*‐axis, lattice parameter *b*) corresponds to the second in‐plane axis (*zigzag*). The [001] direction (*z*‐axis, lattice parameter *c*) is perpendicular to the layer plane and corresponds to the largest lattice parameter, reflecting the distance between van der Waals layers. To be precise, in this notation, the crystal space group should be referred to as *Pcmn*, which includes the same symmetry operations as *Pnma* for a rotated unit cell.

The structure of the investigated samples was confirmed by X‐ray diffraction (XRD) measurements and aberration‐corrected high‐angle annular dark‐field scanning transmission electron microscopy (HAADF‐STEM) combined with energy dispersive X‐ray spectrometry (EDS).

In Figure 1c,d, the results of powder XRD measurements are presented, revealing characteristic reflexes, assigned according to the PDF‐4 database (cards no. 01‐075‐0925 and 96‐153‐7700),^[^ ^41^ ^]^ and allowing to determine the lattice parameters. For SnS the in‐plane parameters *a = *4.318 Å and *b = *3.977 Å, and *c = *11.188 Å, corresponding to the out‐of‐plane direction. For SnSe *a = *4.446 Å, *b = *4.151 Å, and *c = *11.516 Å. Additionally, single crystal XRD measurements were performed, with the incident beam directed either onto the sample surface or its edge, as schematically illustrated in Figure S1 (Supporting Information). For the former, a dominant [001] orientation was observed in the diffraction patterns (Figure S1a,c, Supporting Information), as expected. For the latter (Figure S1b,d, Supporting Information), several weak reflexes were detected, however, none corresponded to the [001] direction, indicating monocrystalline character of the samples, with well‐defined orientation.

In Figure 1d–g the results of the HAADF‐STEM imaging are presented, with clearly visible atomic arrangement, further confirming the high structural quality of the investigated samples. In the insets of panels d and e EDS maps are included, demonstrating homogenous distribution of Sn and S or Se atoms. The corresponding EDS spectra are included in Figure S2 (Supporting Information). In panels f and g the high‐resolution HAADF‐STEM images along [001] direction of the crystals are shown, with indicated spacings between atomic planes (100) and (010), where *d*_100_ = 0.43 nm and *d*_010_ = 0.40 nm for SnS, and *d*_100_ = 0.44 nm and *d*_010_ = 0.42 nm for SnSe. The values remain in good agreement with the lattice parameters *a* and *b*, respectively, determined from the powder XRD measurements.

**Figure 2:**
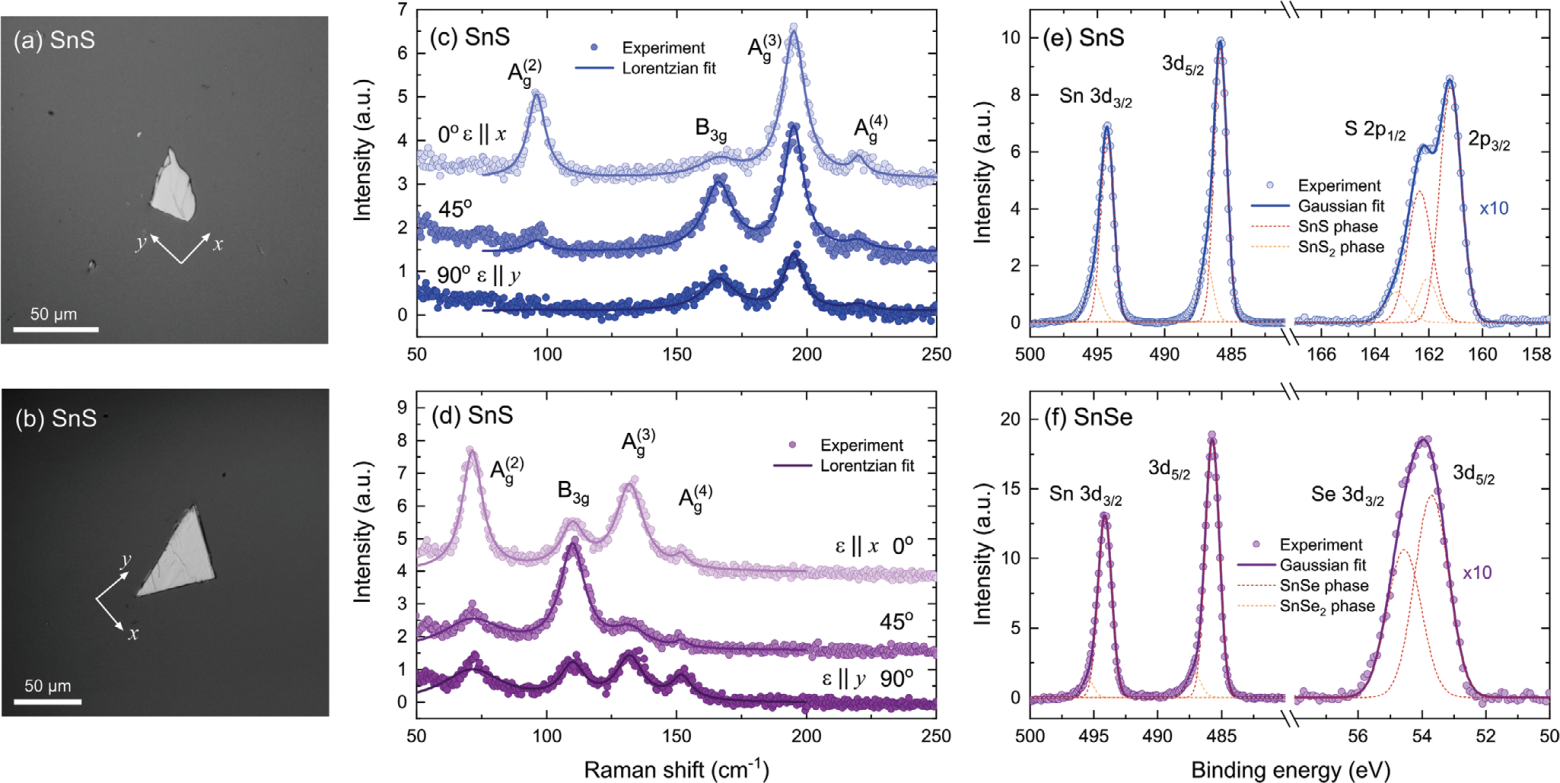
Optical microscope images of the a) SnS and b) SnSe flakes investigated by means of Raman scattering. Polarization‐resolved Raman scattering spectra acquired for c) SnS and d) SnSe. The Lorentzian line‐shapes (solid lines) were fitted to the experimental data (circles), revealing four vibrational modes. The polarization angle of 0° corresponds to the *x* direction of the crystal. Core level XPS spectra of e) SnS and f) SnSe. The Gaussian line‐shapes (dark solid lines) were fitted to the experimental points (circles). The components of the Gaussian fits corresponding to SnX and SnX_2_ phases are plotted with dashed red and yellow lines, respectively.

To further confirm the symmetry of the crystals, Raman scattering measurements were performed for exfoliated flakes transferred onto a silicon substrate, shown in the optical microscope images in **Figure** **2**a,b. For both samples, the Raman spectra (Figure 2c,d) revealed four phonon modes characteristic for the *mmm* point group: three (out of four theoretically predicted) A_g_ and one B_3__g_ mode, corresponding to symmetric and asymmetric shear vibrations, respectively.^[^ ^42^ ^]^ The exact frequencies of the observed modes are summarized in Table S1 (Supporting Information). Investigation of the polarization dependence of the Raman spectra allowed us to determine the orientation of the examined flakes. For the polarization angle of 0°, corresponding to the *x* crystallographic direction, all three A_g_ modes are the most prominent, and their intensity gradually decreases as the polarization angle approaches 90° (*y* direction). The B_3__g_ mode, characterized by four‐fold symmetry, is weak for either *x* or *y* polarization and becomes more pronounced for the intermediate angle of 45°. Such evolution of the spectra is in perfect agreement with previous reports.^[^ ^43^, ^44^, ^45^, ^46^ ^]^

An investigation of the chemical composition was performed using core‐level X‐ray photoemission spectroscopy (XPS). The full‐scale spectra, plotted in Figure S3 (Supporting Information), only show the presence of Sn and S or Se atoms, with negligible traces of oxygen and carbon, indicating high purity of the sample surface. In Figure 2e,f regions of the spectra with characteristic features related to contributing elements are presented for SnS (panel e) and SnSe (panel f), identified after Moulder and Chastain.^[^ ^47^ ^]^ The energies of individual orbital levels were determined by fitting the Gaussian line shape to the acquired data. For S in SnS a doublet corresponding to 2*p* orbitals was observed, with the expected *p*_3/2 _: *p*_1/2_ intensity ratio of ≈2:1. For Se in SnSe, a 3*d* doublet can be identified, with the *d*_5/2_: *d*_3/2_ intensity ratio of ≈3:2. For Sn atoms, signal associated with 3*d* orbitals was detected. The exact energies and spin‐orbit splitting values of the observed lines are summarized in Table S1 (Supporting Information). Along with the core levels corresponding to the SnX phase (with Sn at the +2 oxidation state), plotted as red dashed components of the Gaussian fits, a weak signal originating from the SnX_2_ phase (Sn at the +4 oxidation state, yellow dashed lines) was observed as an asymmetrical broadening of the XPS lines. The contribution of side‐lines could not be resolved for the SnSe Se 3*d* peak due to the close position of *d*_3/2_ and *d*_5/2_ components, but is apparent for other measured lines, indicating the presence of chalcogen‐rich domains at the sample surface, which is not unusual (tin vacancies are one of the most stable native defects in the system, responsible for the intrinsic *p*‐type character).^[^ ^49^, ^50^, ^51^ ^]^

### 2.2 Optical Properties

**Figure 3:**
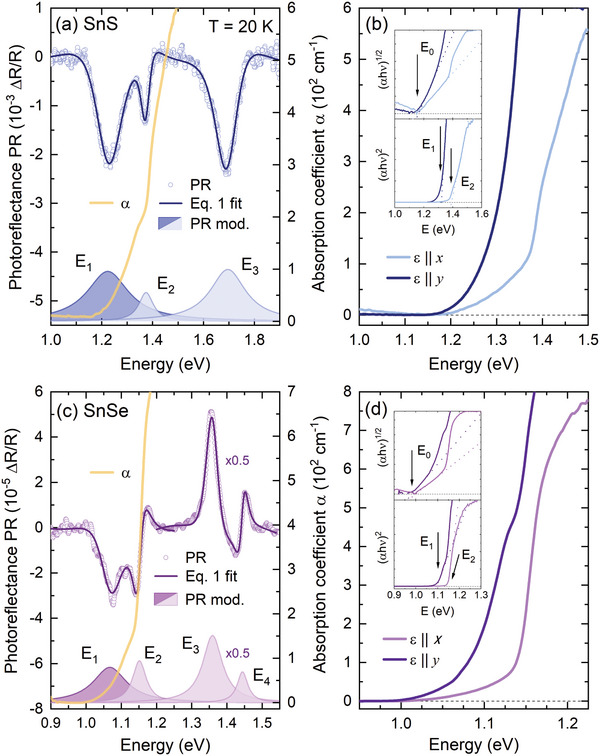
a,c) The unpolarized photoreflectance and optical absorption spectra acquired for SnS (a) and SnSe (c). The fit with the Aspnes formula (dark solid lines) is superimposed over the experimental PR data (circles). The absorption coefficient α is plotted with solid yellow lines. Shaded areas at the bottom of each panel are the PR resonances moduli (Equation 2), with the dark and light color corresponding to *y* and *x* polarization of the optical transition, respectively. In panel c, the *E_3_* and *E_4_* resonances and corresponding moduli are scaled by a factor of 0.5 for clarity. b,d) The optical absorption spectra measured at the incident light polarized along *x* (bright solid lines) and *y* (dark solid lines) crystallographic direction for SnS (b) and SnSe (d). In the insets Tauc plots for indirect and direct absorption edge are shown.

The optical properties of SnS and SnSe crystals were investigated by means of complementary methods of optical spectroscopy: photoreflectance (PR), sensitive to direct optical transitions,^[^ ^52^, ^53^, ^54^ ^]^ and optical absorption, allowing detection of both direct and indirect bandgap. In **Figure** **3**, a comparison of the PR and optical absorption spectra acquired at the temperature of 20 K for SnS (Figure 3a) and SnSe (Figure 3c) is presented. For both materials, in the PR spectra (plotted with blue and purple circles) multiple resonances are visible, indicating the contribution of three and four optical transitions for SnS and SnSe, respectively, labeled *E_1_*–*E_4_* in the figure. In order to determine the energies of the transitions, the Aspnes formula,^[^ ^55^ ^]^ given by

(1) ΔRRℏω=Re∑Ciejφiℏω−Ei+iΓi−2

was fitted to the experimental data. In the equation, *C_i_* is the amplitude of the *i*‐th PR resonance, *φ_i_* is the phase, *Γ_i_* is the broadening and *E_i_* is the energy. The energies, corresponding to the temperature of 20 K are summarized in **Table** **1**. Based on the fitting parameters the resonance moduli, plotted as shaded areas in Figure 3a,c, were calculated, using the formula

**Table 1:** Experimental and theoretical energies and polarizations of measured and predicted optical transitions, along with their assignation to certain BZ points. The experimental values correspond to the temperature of 20 K.

	Transition	BZ point	Polarization	Energy (eV)		
				Experiment (at 20 K)		DFT (at 0 K)
				Absorption	PR	
SnS	E_0_	Γ‐X→ Γ‐Y	–	1.16	–	1.16
	*E*_1_	Γ‐Y	y	1.23	1.23	1.39
	*E*_2_	Γ‐X	x	1.38	1.38	1.75
	*E*_3_	U	x	–	1.70	1.97
SnSe	*E*_0_	Γ‐X→ Γ‐Y	–	0.99	–	0.95
	*E*_1_	Γ‐Y	y	1.09	1.07	1.16
	*E*_2_	Γ‐X	x	1.14	1.15	1.31
	*E*_3_	U	x	–	1.36	1.63
	*E*_4_	Y‐S	x	–	1.45	1.72

(2) $$\Delta \rho_{i}\;\left(E\right)=\frac{\left|C_{i}\right|}{{\left(E-E_{i}\right)}^{2}+\Gamma_{i}^{2}}$$

The area under the modulus curve is related to the transition oscillator strength.

The PR resonances visible in the spectra exhibit strong polarization dependence, as shown by Ho et al.^[^ ^56^ ^]^ and Herninda et al.^[^ ^57^ ^]^ and confirmed by our preliminary results. *E_1_* transition, for both SnS and SnSe, is polarized along the *y* direction, while all the energetically higher features manifest the *x* polarization, as illustrated by different shades of the moduli plots (dark for *y* and light for *x* polarization) corresponding to the measured PR resonances. The optical absorption coefficient spectra (yellow solid lines in Figure 3a,c) exhibit a characteristic step‐like shape, also suggesting the contribution of multiple optical transitions, with the two branches corresponding well to the *E_1_* and *E_2_* PR resonances. Above ≈1.5 eV for SnS and ≈1.3 eV for SnSe the measured signal is saturated and no features attributed to the higher transitions could be observed, however, below the first resonance energy, an absorption tail is present, which may be evidence of the indirect character of the fundamental bandgap. The indirect absorption edge could be better resolved in the spectra acquired for the incident light polarized along one of the main crystallographic directions (i.e., the electric component of the EM field *ε* || *x* or *ε* || *y*), presented in Figure 3b,d. A significant shift of the direct absorption edge with varying polarization can be observed, related to the changes in the probability of individual transitions, in line with the PR resonances polarization dependence. From the Tauc plots^[^ ^58^ ^]^ of the absorption coefficient spectra, presented in the insets of panels b and d, the energies of the individual optical transitions were extracted. For the *x* polarization, the linear region in the (*αhν*)^1/2^ plot is clearly visible, allowing us to determine the fundamental indirect bandgaps *E_0_* of 1.16 eV for SnS and 0.99 eV for SnSe. The (*αhν*)^2^ plots of the energetically higher regions of the spectra for both polarizations provided the energies of direct transitions *E_1_* and *E_2_*, in perfect agreement with PR, as compared in Table 1. Some minor discrepancies may result from the fact that the Tauc plot method is best applicable for materials with simple electronic band dispersion, exhibiting the absorption edge originating from an optical transition between well‐defined parabolic‐like valleys.

**Figure 4:**
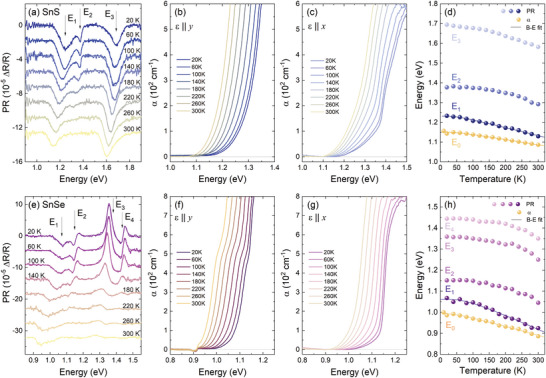
The temperature evolution of the a,e) photoreflectance and b,c,f,g) optical absorption spectra polarized along *y* (b,f) and *x* (c,g) crystallographic directions, acquired for SnS (a,b,c) and SnSe (e,f,g). d,h) Temperature dependence of the optical transition energies determined based on the optical absorption (*E_0_*) and PR (*E_1_ – E_4_*) measurements for SnS (d) and SnSe (h). The experimental data (circles) are approximated by Bose‐Einstein formula (solid lines).

To better understand the optical activity of SnXs, temperature‐dependent experiments were carried out. The obtained results are presented in **Figures** **4** and S4 (Supporting Information). The temperature evolution of the PR spectra (Figure 4a,d) reveals the expected thermal redshift of PR resonance energies and a decrease in their amplitude. In the case of optical absorption, the influence of the temperature was investigated using either unpolarized light (Figure S4a,e Supporting Information) or light polarized linearly along the *x* and *y* direction (Figure 4b,c,g,f, respectively). The energies of the absorption edge were determined from polarized spectra using the Tauc plot method, as shown in Figure S4b–d (Supporting Information). Temperature dependences of *E_0_* and *E_1_* transition energies (Figure 4d,h) were approximated by Bose‐Einstein (B‐E)^[^ ^59^ ^]^ and Varshni^[^ ^60^ ^]^ formulas, providing information about the electron‐phonon interaction strength. The equations and obtained values of the fitting parameters values are given in Table S2 (Supporting Information). Both approaches allow to reproduce the temperature dependence with good accuracy, although the B‐E procedure is more accurate at low temperatures. For the energetically higher transitions, the fits did not converge or provide non‐physical values, which may be related to the uncommon shape of the plots, resulting from the uncertainties of the energies extracted from the PR spectra, especially at higher temperatures.

### 2.3 Electronic Band Structure

**Figure 5:**
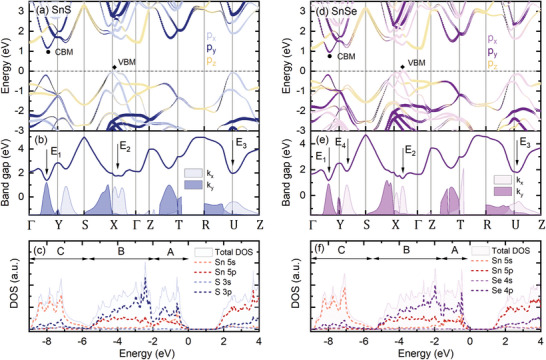
a,d) The electronic band dispersion of SnS (a) and SnSe (d) along high‐symmetry BZ paths with superimposed contribution of the *x*, *y*, and *z* components of the Sn and X *p* orbitals. b,e) The interband distance (solid line) and transition squared matrix element *k_x_* and *k_y_* components (bright and dark shaded areas, respectively). The optical transitions observed in the experiment are assigned to certain BZ points by arrows. c,f) The calculated total (shaded areas) and partial (dashed lines) density of states, corresponding to individual orbitals. The energy scale is relative to the VBM energy (*E_VBM_* = 0). In panels a, b, and c results for SnS are presented, while d, e, and f correspond to SnSe.

Based on the absolute energies and polarizations of the optical transitions observed in the experiment, we propose their assignation to certain Brillouin zone points. In **Figure** **5**a,c the electronic band structure is presented, calculated employing DFT, with the use of Heyd‐Scuseria‐Ernzerhof (HSE06) hybrid functional (for the computational details see *Experimental* Section). As shown in our previous work,^[^ ^40^ ^]^ for SnX crystals the approach allows us to accurately reproduce the band dispersion and provides energies of the fundamental bandgap closest to the experimental values.

The calculations confirm the multivalley character of the band dispersion, with the conduction band minimum (CBM) in the Γ‐Y path and a characteristic shape of the valence band maximum (VBM) in the Γ‐X path, composed of two hole pockets. The band shape has been observed experimentally in the ARPES measurements and strongly influences the thermoelectric efficiency of the materials,^[^ ^40^, ^61^, ^62^ ^]^ The band dispersion is generally similar to germanium monochalcogenides (GeS, GeSe), except for the valence band in the Γ point, pushed toward higher binding energies relative to the VBM.^[^ ^13^, ^14^, ^40^, ^63^ ^]^ Hence, the quasi‐direct bandgap character observed for GeS^[^ ^63^ ^]^ and GeSe^[^ ^13^, ^14^ ^]^ is not present for SnS and SnSe crystals. In Figure 5b,e the relative distance between the highest valence band and lowest conduction band is plotted, along with in‐plane components of the transition squared matrix element (shaded areas), determining the transition oscillator strength and, as a consequence, its probability. The matrix element distribution is governed by the selection rules and indicates for which polarization a transition is allowed. By comparing the experimental results to the calculated band structure we assign the observed optical transitions as labeled in Figure 5b,e and summarized in Table 1. The *E_0_* transition can be attributed to the indirect fundamental bandgap between VBM and CBM. The lowest direct transition *E_1_*, polarized along the *y* direction, corresponds to the valley in the Γ‐Y path, while *E_2_*, active for the *x* polarization, occurs in the Γ‐X path, close to the X point. The transition *E_3_* can be assigned to the U point, and *E_4_*, visible only for SnSe, to a critical point in the Y‐S path. The agreement between experimental and theoretical energies is excellent for *E*_0_, but less satisfactory for the higher‐energy transitions. Such discrepancies may occur in DFT calculations, as the method is limited by inherent approximations and sensitive to computational parameters. While the band shapes are generally well reproduced, the accuracy of absolute energy predictions may be poorer, especially at higher binding energies, where electron‐electron interactions become more pronounced. Therefore, to find a plausible interpretation of the experimental results we focus mainly on the polarization and the matrix element in‐plane components ratio. The anisotropy of the matrix element distribution and, as a consequence, the polarization of the optical transitions is related to the orbital composition of the electronic bands. In Figure 5c,f the density of states (DOS) is plotted, including both total DOS (shaded areas) and partial contribution of Sn and X valence orbitals (dashed lines): Sn 5*s*, 5*p* and S 3*s*, 3*p* or Se 4*s*, 4*p*. According to DFT calculations, also *d* orbitals (often referred to as semi‐core states) have a minor contribution to the total DOS, which is not plotted in the figure for clarity. It was shown that either including the *d* states in the band structure calculation or treating them as core‐levels, provides nearly identical results.^[^ ^19^, ^20^ ^]^ The conduction band of both materials is mainly composed of Sn 5*p* orbitals, with the addition of X *p* states. In the valence band three regions can be distinguished, labeled A, B, and C in Figure 5c,f, as was also shown for other MXs.^[^ ^20^, ^57^ ^]^ The A band is composed of a mixture of Sn 5*s*, 5*p*, and X *p* orbitals, the B region is dominated by X *p* states, with a contribution of Sn *p* orbitals, and the C peak almost entirely consists of Sn 5*s* states. The contribution of the 5*s* orbitals to the upper valence band is a consequence of the presence of stereochemically active *s^2^* lone electron pairs in the crystal structure and will be discussed further in the text. In Figure 5a,d, the contribution of the three spatial components of the *p* orbital (*p_x_*, *p_y_*, *p_z_*), oriented along respective crystallographic axes, is superimposed over the band dispersion. In the figure, *p* states of both Sn and X atoms are combined. A more detailed picture, including also the *s* states and resolving contribution of different elements, is presented in Figure S5 (Supporting Information). In the orbital composition plots the size of the points is proportional to the contribution of an orbital to a certain band. The distribution of the three components across the BZ is particularly interesting regarding the topmost valence and lowermost conduction band, as it affects the optical properties observed in the experiment. In the proximity of the X and U points of the BZ, the bands are mainly composed of *p_x_* orbitals, resulting in high *k_x_* matrix element components, and consequently polarization of the optical transition along the *x* direction. Similarly, in the Γ‐Y and Z‐T paths *p_y_* orbitals dominate, giving rise to high *k*_*y*_ matrix element components and determining the y polarization of the optical transitions. It can also be seen that in the BZ regions where both valence and conduction bands consist mainly of *p_z_* states (Γ, Z, S, and R points), the in‐plane matrix element vanishes, as a result of a small value of the overlap integral. Therefore, in the applied experimental configuration, no optical transition attributed to the Γ point of the BZ was observed, despite relatively low energy with respect to the fundamental bandgap.

### 2.4 Photoemission Study

**Figure 6:**
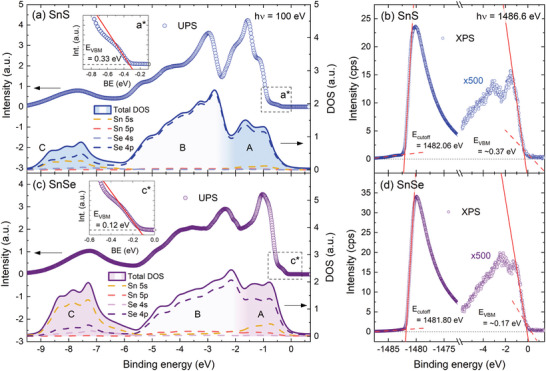
a,c) The results of the UPS measurements (circles, top of each panel) acquired with the use of synchrotron radiation at the excitation energy of 100 eV, compared with the simulated valence band (solid and dashed lines, bottom of each panel) based on the calculated DOS, corrected by the photoionization cross‐section for individual orbitals and broadening of the electronic states. A, B, and C regions of the valence band are labeled and distinguished by different shades of the area under the DOS curve. In the insets the boxed regions of the experimental spectra labeled a^*^ and c^*^ are enlarged to better illustrate the VBM and determine *E_VBM_*. b,d) The low (valence band) and high (secondary electron cut‐off) binding energy regions of the lab‐based XPS spectra (acquired with the use of Al K_α_ line of 1486.6 eV), allowing to extract the *E_cut‐off_* and *E_VBM_* (less accurate than from UPS spectra). The valence band regions of the spectra are scaled by a factor of 500 for better visualization.

The valence band DOS can be experimentally investigated by means of photoemission spectroscopy. In this study we apply two techniques, exploiting different radiation sources and excitation energies. The lab‐based XPS, utilizing the Al K_α_ line of *hν* = 1486.6 eV, allows observation of core‐level states, confirming the material composition and quality, and the high energy secondary electron cut‐off, providing direct information about the work function of the investigated sample. In terms of valence band investigation, for high excitation energies, the photoionization cross‐section of the valence orbitals is relatively small, resulting in low photoemission intensity. Therefore, the second applied method was UV photoemission spectroscopy (UPS), exploiting monochromatic synchrotron radiation of *hν* = 100 eV. The technique and the experimental setup provide significantly improved resolution and sensitivity, along with the possibility of angle‐resolved measurements, which are reported in our previous work.^[^ ^40^ ^]^ Both techniques allow measurements of the valence band, however, UPS is more suitable, considering the photoionization cross‐section of the valence orbitals up to two orders of magnitude higher for the excitation energy of 100 eV compared to 1.5 keV.^[^ ^64^, ^65^ ^]^ In **Figure** **6**a,c the UPS spectra (top plot of each panel) are compared with the simulated valence band (bottom plots), obtained by applying the following procedure to the calculated partial DOS: first, the contribution from the orbitals was weighted using the photoemission cross‐sections corresponding to the excitation energy of 100 eV, according to Yeh and Lindau.^[^ ^64^ ^]^ Next, to introduce the broadening of the electronic states, the partial DOS was convolved with Lorentzian (natural broadening) and Gaussian (thermal broadening) functions. The UPS spectra were corrected by subtracting the background originating from the inelastic electron scattering. The raw, uncorrected spectra are plotted in Figure S6 (Supporting Information). It can be seen for both SnS and SnSe that after taking into account the photoionization cross‐section, the signal from the A and B bands is strongly dominated by X *p* orbitals, and the contribution of Sn 5*s* states is distinct only in the C band. The simulated curves are in perfect agreement with the experimental spectra in the B and C regions but diverge in the A region. In the calculations, the intensity of the A band is lower with respect to the B band, but in the experiment, we observe the opposite relation. The discrepancy may result from the fact that in the measurement light polarized linearly was used, interacting differently with the three spatial components of *p* orbitals. Effectively, the photoionization cross‐section for *p* states might be smaller than predicted, while estimated correctly for s orbitals, insensitive to the polarization. Then, the contribution of the Sn 5*s* states to the photoemission from the A region should be greater, resulting in higher overall detected intensity. Another factor that may influence the respective band's intensity ratio is the possibility that DFT calculations underestimate the contribution of the Sn 5*s* states to the topmost region of the valence band. The XPS spectra acquired for SnS and SnSe, visualizing the secondary electron cut‐off and valence band maximum are presented in Figure 6b,d. From the extrapolation of the linear region at the high binding energy side of the spectrum, the cut‐off energy *E_cut‐off_* was determined. The valence band maximum energy *E_VBM_* can also be estimated (with respect to the Fermi level *E_F_*), however considering the spectral resolution and intensity of the signal originating from the valence band, more reliable and precise values of *E_VBM_* are extracted from the UPS data, as shown in the insets of Figure 6a,c. It can be noticed that the VBM is observed at slightly higher energies in the XPS measurements. Such a shift can be explained by the fact that the DOS at VBM (close to the X point of the BZ) is low and cannot be properly resolved in the XPS measurements. In the UPS spectra, a characteristic step attributed to the VBM is clearly visible, followed by a rapid rise of the photoemission signal originating from a band in the Γ point.

The values of *E_VBM_* obtained for SnS and SnSe are 0.33 and 0.12 eV, respectively. The material work function (*φ*) can be calculated from the relation.

(3) $$\varphi =h\nu -E_{cut-off}$$

**Figure 7:**
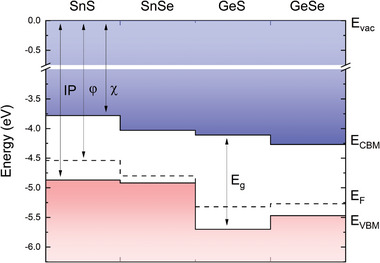
The band alignment diagram for the group IV‐IV chalcogenides SnS, SnSe, GeS, and GeSe, with respect to vacuum energy *E_vac_*. The values for GeX crystals are adapted after Grodzicki et al.^[^ ^66^ ^]^

Then, the ionization potential (*IP*) is given by

(4) $$IP=\varphi +E_{VBM}$$

and the electron affinity (χ) can be defined as

(5) $$\chi =IP-E_{g}$$

where *E_g_* is the fundamental energy gap determined from the optical absorption measurements. The values of the above parameters for SnS and SnSe are summarized in **Table** **2** and **Figure** **7**. Since *φ*, *IP*, and *χ* are parameters particularly important for the integration of different materials, the values acquired for SnXs are compared with GeS, and GeSe, belonging to the same family of orthorhombic group IV monochalcogenides and investigated in our previous work.^[^ ^66^ ^]^

**Table 2:** Ionization potential, work function, electron affinity, and room temperature energy gap determined for SnS, SnSe, GeS, and GeSe. The values for GeX crystals are adapted after Grodzicki et al.^[^ ^66^ ^]^

	*IP* [(eV)]	*φ* [(eV)]	*χ* [(eV)]	*E_g_* [(eV)]
SnS	4.87	4.54	3.78	1.09
SnSe	4.92	4.80	4.03	0.89
GeS	5.70	5.32	4.11	1.59
GeSe	5.47	5.27	4.27	1.20

It should be noted that the work function, related to the Fermi level position inside the bandgap, may vary depending on the intrinsic defects or intentional dopants concentration, therefore the parameter more suitable for comparison with other studies is the *IP*, i.e., the VBM position with respect to the vacuum level (*E_vac_*).

**Figure 8:**
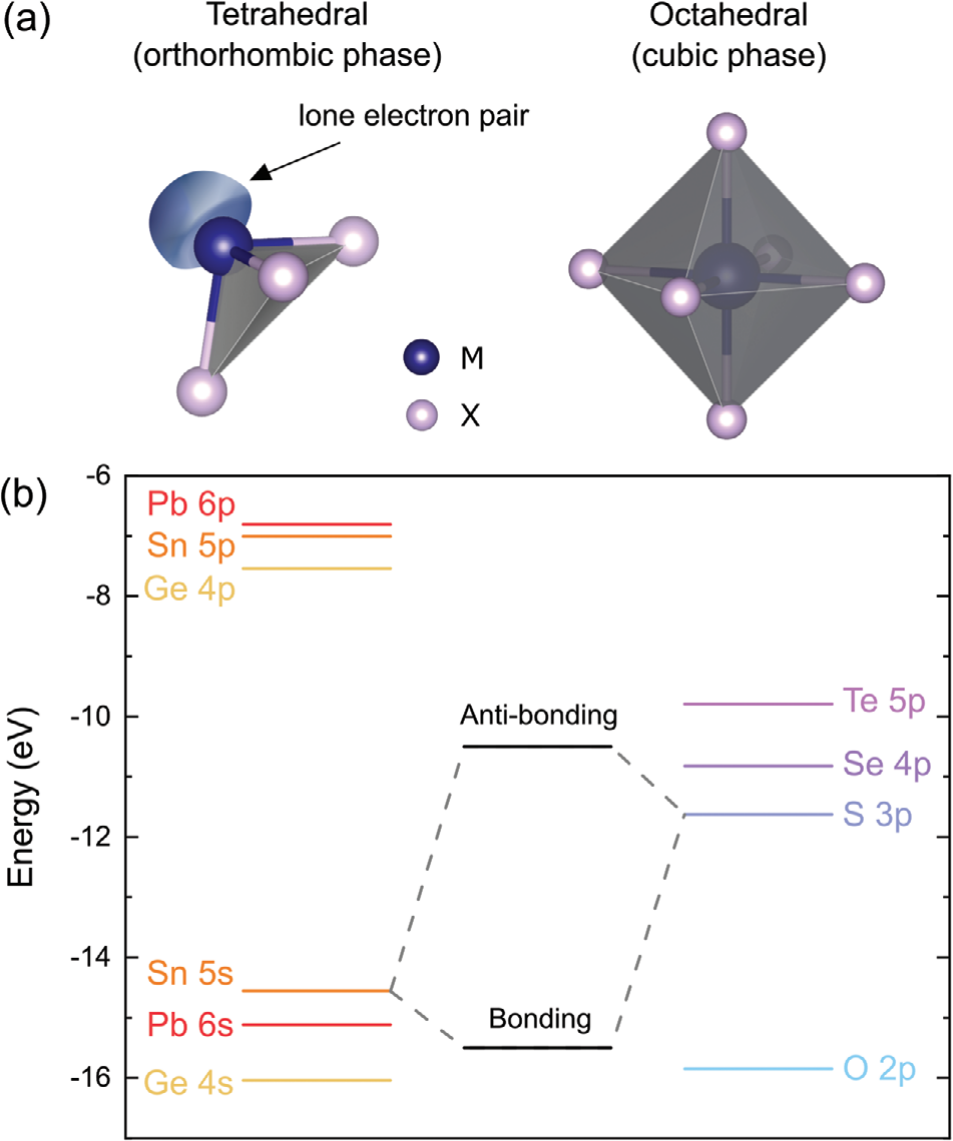
a) Schematic illustration of the tetrahedral (left) and octahedral (right) coordination of metal atoms in the orthorhombic and cubic phases of MXs, respectively. b) Diagram of the electronic configuration energies of the atomic valence *s* and *p* orbital for group IV elements Ge, Sn, and Pb, and group VI elements O, S, Se, and Te. The orbital energies are adapted after Mann et al.^[^ ^68^ ^]^ The hybridized bonding and anti‐bonding states are only presented for the illustration of the phenomenon and do not correspond to the values on the energy scale.

The obtained values of the *IP* are relatively low compared to other semiconducting van der Waals crystals, which leads to some significant consequences considering potential applications, such as band alignment with window layers for photovoltaics, affecting the device efficiency, or with metals for electrical contacts, determining the ohmic or Schottky character of the junction. The reason for this is the aforementioned contribution of the metal *s* ^2^ lone electron pairs to the high energy region of the valence band, confirmed by previous studies.^[^ ^19^, ^67^ ^]^ In all MXs the metal atom is in the +2 oxidation state. For group IV metals it implies either tetrahedral or octahedral coordination, illustrated in **Figure** **8**a. The former is characterized by three bonds with chalcogen atoms and one lone electron pair in the fourth vertex of the tetrahedron, forming a distorted orthorhombic crystal lattice. In the latter, six M─X bonds result in a perfectly symmetric rocksalt structure.^[^ ^18^, ^21^ ^]^ The lattice distortion is related to the stereochemical activity of the *s* ^2^ lone pairs, observed for GeS, GeSe, GeTe, SnS, and SnSe, but not for other group IV‐VI compounds with the same stoichiometry, such as SnTe, PbS, PbSe, and PbTe. The phenomenon can be partly explained by the revised lone pair model proposed by Walsh et al.^[^ ^21^ ^]^ In the considered materials the metal valence *s* orbitals hybridize with chalcogen *p* states, forming bonding and anti‐bonding states (as schematically illustrated in Figure 8b), contributing to the high (C band) and low (A band) binding energy regions of the valence band, respectively. The amount of the contribution of the M *s* orbitals to the anti‐bonding state is determined by the relative energy of the M *s* and X *p* levels, yielding higher contribution for smaller energy distance. For the strong M *s* component, the interaction of the unoccupied M *p* orbitals with the anti‐bonding state leads to asymmetric electron density distribution with a directional lone electron pair. When M *s* contribution is minor, the interaction is weak, and stereochemically active lone pairs do not form. The relative positions of the M *s* and X *p* valence levels for group IV and VI elements are presented in a diagram in Figure 8b, with the energies adapted after Mann et al.^[^ ^68^ ^]^ It can be seen that due to the lowest energy distance, the most prominent lone pairs form for oxides, and the X *p* energy increases down the VI group. For metal atoms, the Sn 5*s* state is positioned higher relative to both Ge 4*s* and Pb 6*s* levels, reducing the effective distance to the X *p* states, which explains the significantly lower *IP* observed for SnXs compared to GeXs. Considering only tin compounds, we should expect stronger mixing of the Sn 5*s* orbitals with S 3*p* than with Se 4*p* states, and therefore greater contribution to the A band for SnS. In,

Figure S7 (Supporting Information) the calculated Sn 5*s* contribution to the total DOS is plotted for both investigated materials, confirming the predictions.

The revised lone pair model is in agreement with the experimental results obtained for SnX crystals, however does not explain the formation of the stereochemically active lone pairs in GeX compounds and their absence in PbXs. An attempt to justify the phenomenon was made by Smiles et al.,^[^ ^20^ ^]^ who proposed that the atomic radius and bond lengths may affect the resulting favorable structure. The theoretical study performed by the authors for GeS and GeSe, simulating the influence of the bond length on the optimized geometry by varying the unit cell volume, did not resolve the issue, however other evidence that the explanation might be valid can be found in the literature. XRD studies of the pressure‐induced structural phase transition of PbX crystals revealed a transformation from a perfectly symmetric rock‐salt to a distorted orthorhombic structure with space group *Cmcm* (a supergroup of *Pnma*).^[^ ^69^, ^70^, ^71^ ^]^ Furthermore, in the investigations of the thermal expansion of GeX materials, a phase transition from the orthorhombic (GeSe) or rhombohedral (GeTe) to the cubic system was observed.^[^ ^72^, ^73^ ^]^ GeS did not undergo any phase transition up to the melting point, indicating superior stability of the distorted structure.^[^ ^73^ ^]^ The observed behavior of both Pb and Ge chalcogenides is in line with the hypothesis that the interatomic distance may influence the stereochemical activity of the lone electron pairs. In the case of the former, increasing the hydrostatic pressure leads to an effective reduction of the interatomic distance and induces stronger interaction between Pb and X orbitals, resulting in the crystal lattice distortion. For the latter, the thermal expansion causes an opposite effect, weakening the interaction and observed as a phase transition to a structure with higher symmetry. Considering the Sn chalcogenides, it is worth mentioning that in the high‐temperature regime (above ≈800 K) the *Cmcm* phase appears, although the transition is only related to the relative change of the lattice parameters (at the transition temperature *a* = *b*, resulting in higher symmetry of the structure) and does not involve major atomic rearrangement.^[^ ^74^ ^]^ The result confirms the trends predicted based on the atomic orbital energies, regarding the relation between the stability of the *s^2^* lone pairs and the energy distance between M *s* and X *p* valence states.

## 3 Conclusion

In conclusion, the optical properties of SnS and SnSe van der Waals crystals were investigated by complementary methods of optical spectroscopy (modulated reflectance and optical absorption) combined with ab initio calculations of the electronic band structure and density of states. The experimental studies confirmed the predicted indirect character of the fundamental bandgaps of SnS (1.09 eV at room temperature) and SnSe (0.89 eV), and revealed strong linear dichroism of the energetically higher direct optical transitions. The anisotropy of the optical properties originates from the electronic band structure and orbital composition of the individual bands, determining the selection rules and transition probability dependent on the incident light polarization angle.

By means of UV and X‐ray photoemission spectroscopy, the valence bands of SnS and SnSe were examined experimentally, remaining in good agreement with the simulations based on the calculated density of states corrected by the photoionization cross‐section of the individual orbitals. The measurements also allowed us to determine the work functions, ionization potentials, and electron affinities of the materials, providing information about the band alignment with other semiconductors for heterostructure engineering. The obtained relatively low values of the ionization potential (4.87 and 4.92 eV for SnS and SnSe, respectively) can be attributed to the presence of the stereochemically active Sn 5*s* lone electron pairs in the crystal structure. Their formation is governed by the interaction between the unoccupied Sn 5*p* orbitals and the anti‐bonding state of hybridized Sn 5*s* and S or Se valence *p* orbitals. Along with SnX, we discuss the phenomenon and its consequences also for other group IV monochalcogenides.

In general, our results explain the origins of the linear dichroism of the optical properties of SnS and SnSe and point toward potential applications in polarization‐sensitive photodetectors, but also possibilities of integration with other crystals to form van der Waals heterostructures.

## 4 Experimental Section

### Experimental Details—Samples

The investigated samples were SnS and SnSe single crystals synthesized using a modified Bridgman technique using 6N Sn, S, and Se precursors. Prior to synthesis, as received precursors were purified using float zone technique to reach 6N purity. The stoichiometric ratio of Sn and chalcogen were mixed in nugget form into a Bridgman ampoule with a sharp tip to limit nucleation density and enable single crystal formation. The typical growth temperature was set above the melting point of SnS and SnSe, usually at 950 °C. The crystallization occurred by lowering a rate of 1 mm per week from 950 °C hot zone to 300 °C cold zone.

For characterization with different experimental techniques, samples of adequate dimensions were selected and additional preparation procedures were applied, specified further in this section.

### Experimental Details—Structural Characterization

X‐ray diffraction (XRD) measurements were performed on a Marvel Panalytical Empyrean diffractometer in a Bragg‐Brentano configuration using a Cu K_α1_ *λ* = 1.540598 Å x‐ray tube and a Pixcel3D detector. The experiment was conducted under normal conditions and included powder XRD and single crystal measurements, with an X‐ray beam directed either on the sample surface or its edge (perpendicularly to the surface), as illustrated in Figure S1 (Supporting Information). For the surface measurements, a small piece of bulk crystal was placed on a Si/SiO_2_ substrate. For the edge configuration, the same flake was mounted vertically between two substrates. Due to the small sample thickness and roughness of the edges, the detected signal was significantly weaker compared to powder, and surface measurements and positions of the observed reflexes may be less accurate.

The characterization by means of high‐angle annular dark‐field scanning electron microscopy (HAADF‐STEM) and energy dispersive X‐ray spectrometry (EDS) was performed with the use of the state‐of‐the‐art Thermo Fisher Scientific TITAN 60–300 G2 cube microscope, equipped with a super‐X 4 detectors X‐ray spectrometer, operating at an accelerating voltage of 300 kV. For TEM sample preparation, mechanically exfoliated flakes were first subjected to ultrasonication in IPA for 10 min, followed by drop casting onto Au holey support film‐coated TEM grids (Quantifoil UltrAuFoil). The as‐prepared grids were then dried under IR‐lamp for 4 h and exposed to 6 min plasma cleaning in Fischione 1020 plasma cleaner (25% O2, & 75% Ar) before loading into TEM chamber. The experiment was carried out under ultrahigh‐vacuum conditions at room temperature. For HAADF‐STEM imaging, specific parameters including a beam convergence semiangle of 21.4 mrad, a probe beam current of 40 pA, and detector collection angles ranging from 50.5 to 200 mrad were applied.

Raman scattering measurements were performed using a custom‐built optical setup comprising a 550 mm focal‐length grating monochromator coupled with a liquid nitrogen‐cooled CCD array detector. A continuous‐wave (CW) 532 nm laser was used for sample excitation, with an incident power of 200 µW and a focused laser spot size of ≈5 µm. The laser was focused using a 50 × objective lens (numerical aperture, NA = 0.55), which also facilitated signal collection in the backscattering geometry. All experiments were conducted under ambient conditions.

For polarization‐resolved measurements, a Glan‐Taylor calcite linear polarizer and a half‐wave plate were placed in the optical path before the sample, while a second polarizer (analyzer) was used for selective detection. The incident light polarization was adjusted by rotating the half‐wave plate, with the analyzer simultaneously rotated to maintain a parallel configuration (the detected light polarization matched the incident light polarization).

Raman scattering measurements were performed for thin flakes exfoliated from larger crystals and transferred onto a silicon substrate. The lateral dimensions of the flakes were ≈40 µm.

### Experimental Details—Optical Spectroscopy

For the optical spectroscopy measurements (photoreflectance and optical absorption) the samples were exfoliated to obtain a clear surface and mounted on a brass carrier, ensuring good thermal contact, inside a cryostat coupled with a closed‐cycle helium cryocooler. For optical absorption, flakes of 20–50 µm thickness were separated from larger crystals, for which the photoreflectance measurements were performed. The experiments were carried out with the use of dedicated optical setups, composed of a quartz tungsten halogen lamp (a probing white light source), a 550 mm focal length grating monochromator and a detection system, including Si or InGaAs photodiode, and a lock‐in amplifier (Stanford Research Systems SR830). For the photoreflectance measurements the modulation was achieved by periodic excitation with a 532 nm CW laser, mechanically chopped at the frequency of ≈300 Hz. For polarization‐resolved optical absorption measurements, a Glan‐Taylor calcite linear polarizer and an achromatic half‐wave plate were placed in the optical path.

### Experimental Details—Photoemission Spectroscopy

The photoemission studies were performed using two techniques, exploiting different radiation sources. For both variants, the samples were exfoliated under ultra‐high vacuum conditions inside a preparation chamber. The investigated samples were bulk crystals, however considering the penetration depth of the UV and X‐ray radiation in the order of ≈10 nm and the spot size of ≈20 µm, the effectively probed region was confined to only a small portion of the material.

UV photoemission spectroscopy experiments were carried out with the use of the synchrotron radiation (excitation energy of 100 eV) of the URANOS beamline at SOLARIS National Synchrotron Radiation Centre (Kraków, Poland).^[^ ^75^ ^]^ The photoemission signal was detected with a Scienta‐Omicron DA30‐L electron analyzer. The measurements were performed at the temperature of 77 K (achieved in a flow‐type liquid nitrogen cryostat) and base pressure below 5 × 10^−11^ mbar. X‐ray photoemission spectroscopy (including core‐level XPS) exploiting monochromatic Al K_α_ line (1486.6 eV) was carried out at room temperature. In this technique, the photoelectrons were detected with a hemispherical analyzer Argus CU.

The synchrotron‐based UPS and lab‐based XPS techniques were combined to simultaneously determine the valence band maximum energy (*E_VBM_* ​) and the secondary electron cut‐off energy (*E_cut‐off_*). The experimental setup for synchrotron‐based measurements was specifically optimized to detect photoelectrons with relatively high kinetic energies, and the secondary electron cut‐off, originating from low‐energy electrons, typically falls below the detection threshold of this setup.

To address this limitation, commonly a negative sample bias is applied, shifting the cut‐off toward higher kinetic energies and bringing it within the measurable range. While this approach was successfully implemented in the XPS measurements, the synchrotron‐based setup does not support this functionality.

### Computational Details

Ab initio calculations were performed within the framework of density functional theory (DFT) with the use of the relativistic projector‐augmented waves (PAW) datasets^[^ ^76^ ^]^ in Vienna Ab Initio Simulation Package (VASP)^[^ ^77^ ^]^ The Perdew‐Burke‐Ernzerhof (PBE) parametrization of generalized gradients approximation (GGA) to the exchange‐correlation functional was employed.^[^ ^78^ ^]^ Monkhorst‐Pack k‐point grid of 12 × 12 × 3, plane wave energy cutoff of 600 eV, and a semi‐empirical DFT‐D3 correction for vdW interactions were used.^[^ ^79^ ^]^ The electronic band structures were calculated with the use of the Heyd‐Scuseria‐Ernzerhof (HSE06) hybrid functional.^[^ ^80^ ^]^

## Conflict of Interest

The authors declare no conflict of interest.

## Supporting information



Supporting Information

## Data Availability

The data that support the findings of this study are openly available in Zenodo at https://doi.org/10.5281/zenodo.12921997, reference number 12921997.
