# Supplementary material for: Linear Dichroism of the Optical Properties of SnS and SnSe Van der Waals Crystals
Source: Small. 2025 Feb 21;21(12):2410903. doi: 10.1002/smll.202410903 (PMC11947518; doi:10.1002/smll.202410903)
Supplement: Supplementary file 1 — Supporting Information [file SMLL-21-2410903-s001.pdf]

NANO · MICRO
small

Supporting Information

for *Small*, DOI 10.1002/smll.202410903

Linear Dichroism of the Optical Properties of SnS and SnSe Van der Waals Crystals

Agata K. Tołłoczko, Jakub Ziembicki, Miłosz Grodzicki, Jarosław Serafińczuk, Marcin Rosmus, Natalia Olszowska, Sandeep Gorantla, Melike Erdi, Seth A. Tongay and Robert Kudrawiec*

Supporting Information

Linear dichroism of the optical properties of SnS and SnSe van der Waals crystals

Agata K. Tołłoczko,[*a] Jakub Ziembicki,[a] Miłosz Grodzicki,[ab] Jarosław Serafińczuk,[ab] Marcin Rosmus,[c] Natalia Olszowska,[c] Sandeep Gorantla,[b] Melike Erdi,[d] Seth A. Tongay,[d] and Robert Kudrawiec[ab]*

[a] Department of Semiconductor Materials Engineering,
Wrocław University of Science and Technology,
Wybrzeże Wyspiańskiego 27, 50-370 Wrocław, Poland

[b] Łukasiewicz Research Network – PORT Polish Center for Technology Development,
Stabłowicka 147, Wrocław, Poland

[c] Solaris National Synchrotron Radiation Centre, Jagiellonian University, Czerwone Maki 98, 30-392 Kraków, Poland

[d] School for Engineering of Matter, Transport and Energy Arizona State University
Tempe, AZ 85287, USA

*Corresponding author.

E-mail address: agata.tolloczko@pwr.edu.pl

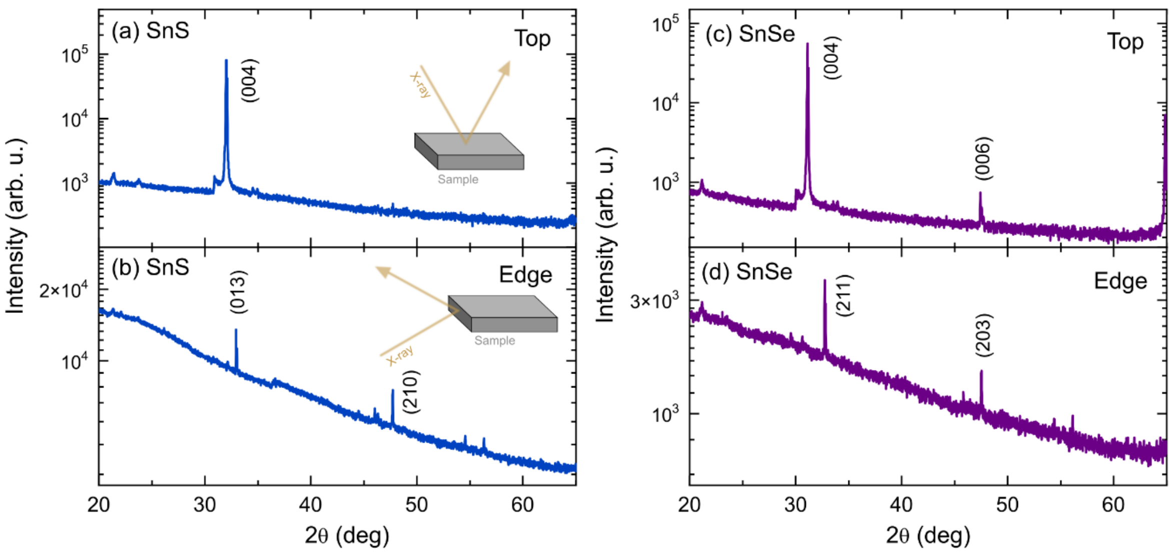

Figure S1. The results of single crystal XRD measurements, acquired for (a,b) SnS and (c,d) SnSe. The incident radiation was directed on the (a,c) sample surface, or (b,d) its edge, as presented in the insets of panels a and b.

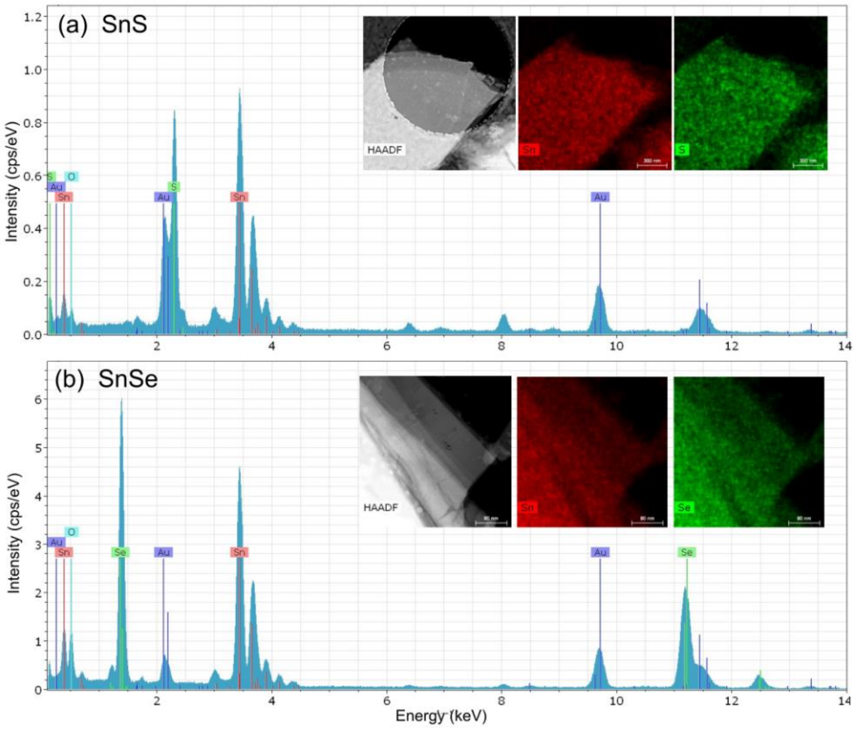

Figure S2. The EDS spectra acquired for (a) SnS and (b) SnSe flakes. The spectra correspond to the EDS maps presented in the insets of each panel.

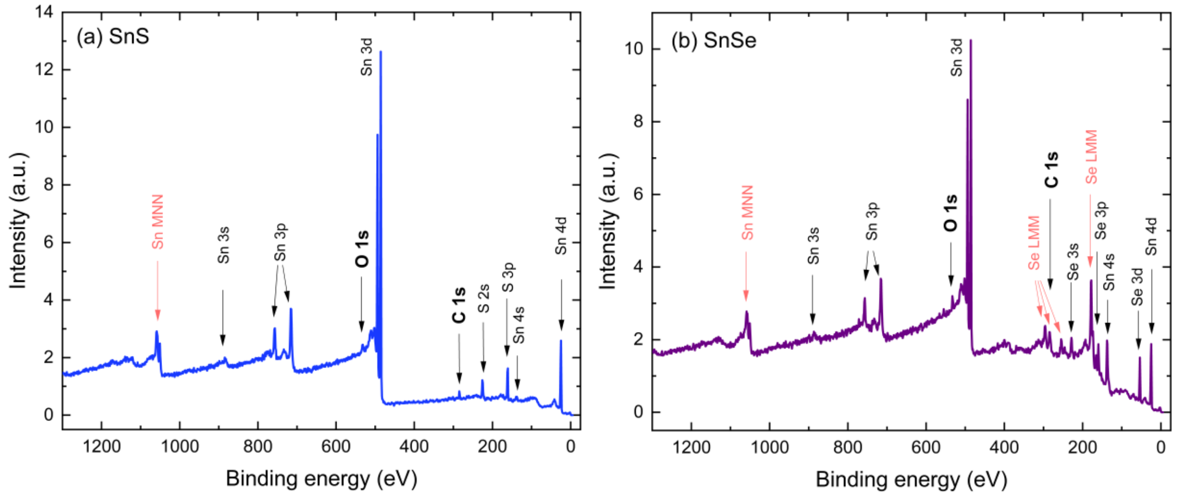

Figure S3. Full-scale core-level XPS spectra acquired for SnS (a) and SnSe (b). Lines corresponding to individual atomic orbitals are identified. Auger lines are marked with red arrows and labels. Along with the signal originating from Sn and S or Se, weak contribution of O 1s (at the biding energy of 532.5 eV) and C 1s (284.7 eV) states was observed. The spectra are interpreted with regard to Moulder and Chastain [S1].

Table S1. Raman modes frequencies and binding energies of the core-level XPS lines (with SO splitting given in parentheses) measured for SnS and SnSe.

	SnS	SnSe
Raman mode	Frequency (cm^{-1})	
$A_g^{(2)}$	96	71
B_{3g}	167	110
$A_g^{(3)}$	195	132
$A_g^{(4)}$	220	152
XPS line	Binding energy line (SO splitting) (eV)	
S $2p_{3/2}$	161.2 (1.1)	-
Sn $3d_{5/2}$	485.8 (8.5)	485.7 (8.4)
Se $3d_{5/2}$	-	53.7 (0.9)

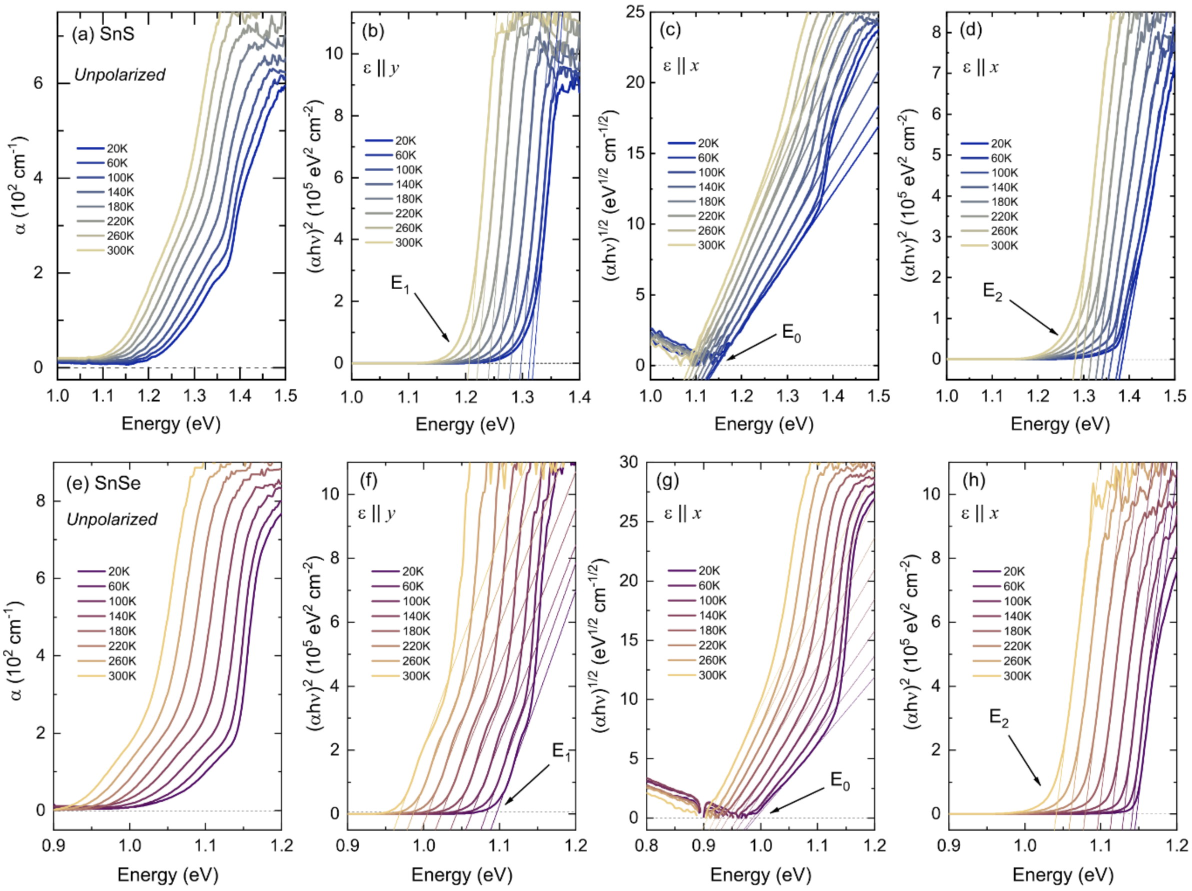

Figure S4. Temperature dependence of the optical absorption measured with unpolarized incident light for (a) SnS and (b) SnSe. The Tauc plots of the (b,d) *y*- and (c,d,g,h) *x*-polarized spectra, allowing to determine the fundamental indirect band gap E_0 and direct transitions energies E_1 and E_2.

Analysis of the optical transition energy temperature dependence

Bose-Einstein formula [S2]

$$E(T) = E(0) - \frac{2a_B}{\exp\left(\frac{\theta_B}{T}\right) - 1} \qquad \text{(Eq. S1)}$$

$E(0)$ - energy at the temperature of 0 K,

a_B - electron-phonon interaction strength,

θ_B - average phonon temperature.

Varshni formula [S3]

$$(T) = E(0) - \frac{\alpha T^2}{\beta + T} \qquad \text{(Eq. S2)}$$

$E(0)$ - energy at the temperature of 0 K,

α, β - semi-empirical Varshni coefficients.

Table S2. Temperature coefficients of the energies of the fundamental indirect (E_0) and lowest direct (E_1) optical transitions, determined from fitting the dependencies with Bose-Einstein (Eq. S1) and Varshni (Eq. S2) formulas.

	Transition	Bose-Einstein			Varshni		
		$E(0)$ (eV)	a_B (meV)	θ_B (K)	$E(0)$ (eV)	α (10^{-4} eV K^{-1})	β (K)
SnS	E_0	1.15	19.2	140	1.15	3.02	110
	E_1	1.23	27.7	133	1.23	4.67	112
SnSe	E_0	0.99	28.4	133	0.99	4.85	119
	E_1	1.06	78.6	223	1.07	8.75	230

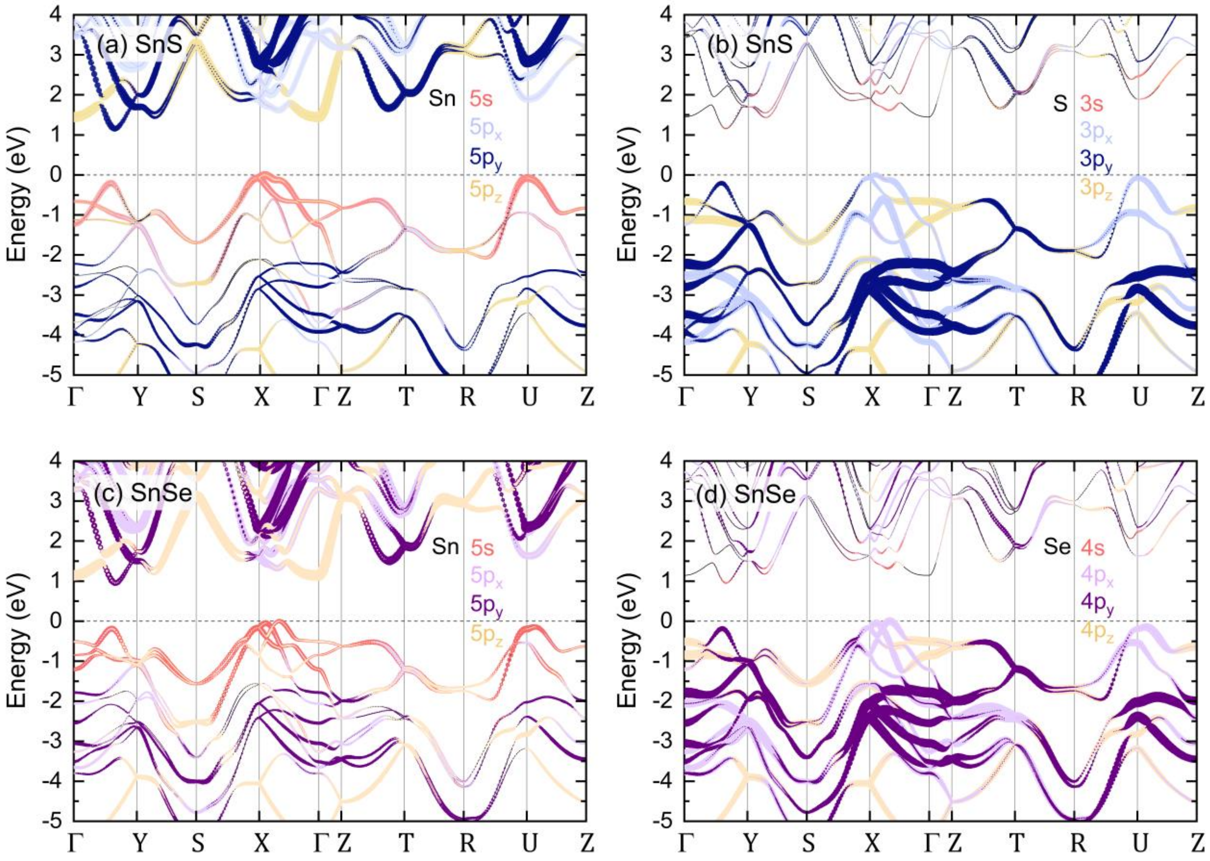

Figure S5. The electronic band structure calculated with the use of mBJ exchange potential for SnS (a,b) and SnSe (c,d), with superimposed contribution of the valence *s* and *p* (with three spatial components p_x, p_y, p_z) orbitals of Sn (a,c), S (b) and Se (c) atoms, illustrated with the size of the plot points.

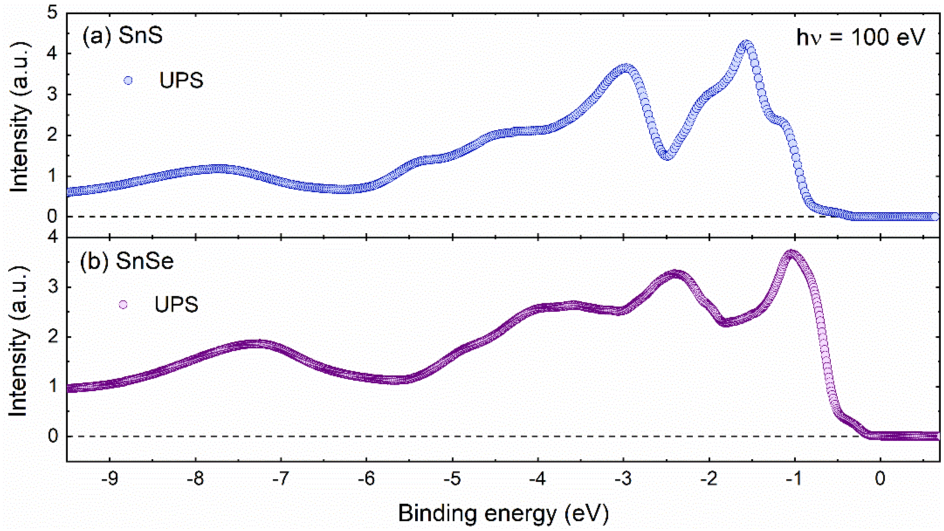

Figure S6. Raw, uncorrected UPS spectra acquired for (a) SnS and (b) SnSe.

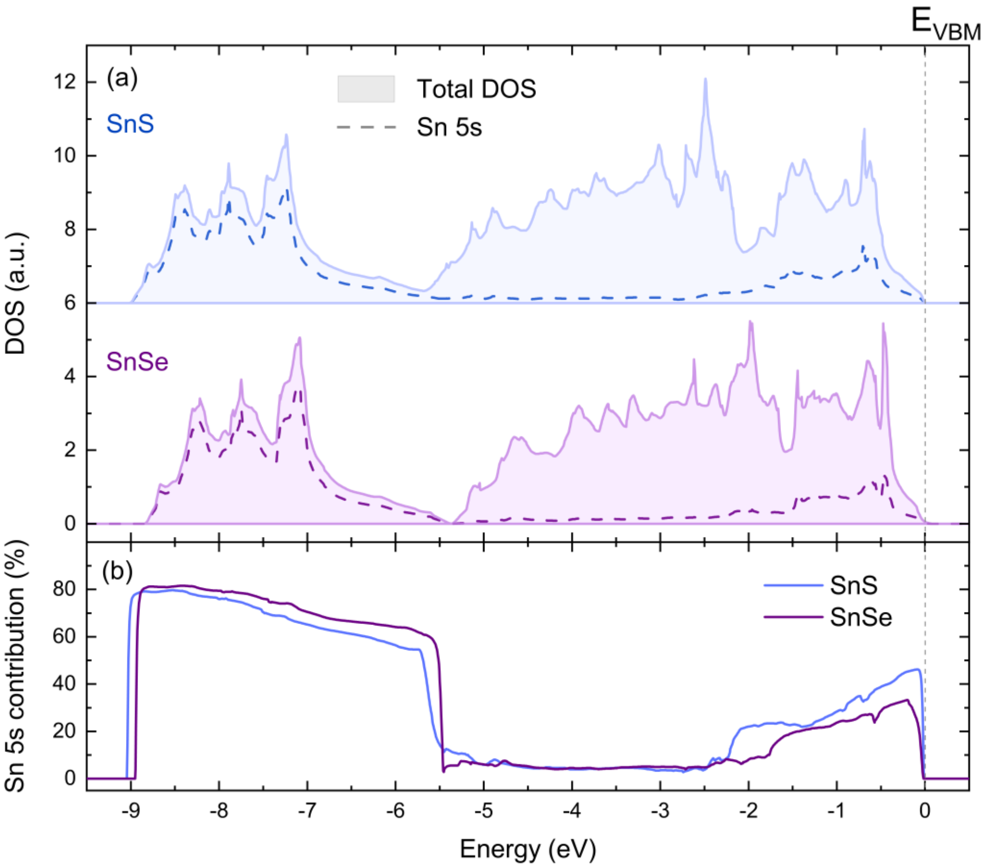

Figure S7. (a) Total (shaded areas) and partial Sn 5*s* (dashed lines) DOS calculated for SnS (top plot) and SnSe (bottom plot). (b) The percentage contribution of the Sn 5*s* orbital to total DOS.

References

[S1] J. F. Moulder and J. Chastain, *Handbook of X-ray Photoelectron Spectroscopy: A Reference Book of Standard Spectra for Identification and Interpretation of XPS Data*, Physical Electronics Division, Perkin-Elmer Corporation, 1992.

[S2] L. Viña, S. Logothetidis and M. Cardona, Phys. Rev. B, 1984, 30, 1979–1991.

[S3] Y. P. Varshni, Physica, 1967, 34, 149–154.
